# Thoracoscopic primary repair is useful for esophageal atresia with tracheoesophageal fistula in neonates with low body weight

**DOI:** 10.1007/s00383-024-05724-x

**Published:** 2024-06-03

**Authors:** Yousuke Gohda, Hiroo Uchida, Chiyoe Shirota, Takahisa Tainaka, Wataru Sumida, Satoshi Makita, Miwa Satomi, Akihiro Yasui, Yoko Kanou, Yoichi Nakagawa, Daiki Kato, Takuya Maeda, Yaohui Guo, Jiahui Liu, Hiroki Ishii, Kazuki Ota, Akinari Hinoki

**Affiliations:** 1 Department of Pediatric Surgery, Nagoya University Graduate School of Medicine, 65 Tsurumai-cho, Showa-ku, Nagoya, 466-8550 Japan; 2 Department of Rare/Intractable Cancer Analysis Research, Nagoya University Graduate School of Medicine, 65 Tsurumai-cho, Showa-ku, Nagoya, 466-8550 Japan

**Keywords:** Anastomotic leakage, Esophageal atresia with tracheoesophageal fistula, Low body weight, Neonates, Thoracoscopic primary repair

## Abstract

**Purpose:**

The surgical indication of thoracoscopic primary repair for esophageal atresia with tracheoesophageal fistula is under debate. The current study aimed to investigate the outcome of thoracoscopic primary repair for esophageal atresia with tracheoesophageal fistula in patients weighing < 2000 g and those who underwent emergency surgery at the age of 0 day.

**Methods:**

The surgical outcomes were compared between patients weighing < 2000 g and those weighing > 2000 g at surgery and between patients who underwent surgery at the age of 0 day and those who underwent surgery at age ≥ 1 day.

**Results:**

In total, 43 patients underwent thoracoscopic primary repair for esophageal atresia with tracheoesophageal fistula. The surgical outcomes according to body weight were similar. Patients who underwent surgery at the age of 0 day were more likely to develop anastomotic leakage than those who underwent surgery at the age of ≥ 1 day (2 vs. 0 case, *p* = 0.02). Anastomotic leakage was treated with conservative therapy.

**Conclusion:**

Thoracoscopic primary repair is safe and useful for esophageal atresia with tracheoesophageal fistula even in newborns weighing < 2000 g. However, emergency surgery at the age of 0 day should be cautiously performed due to the risk of anastomotic leakage.

## Introduction

Esophageal atresia with or without tracheoesophageal fistula (EA/TEF) is a congenital digestive system disorder that occurs in approximately 1 in 3500 to 1 in 4500 newborns [1, 2]. The radical treatment for EA/TEF is esophageal anastomosis. If the gap between the proximal and distal segments of the esophagus is long, which is often observed in gross types A or B, the esophageal pouch should be elongated. Conversely, if the gap is not long, as is often the case in gross type C, primary anastomosis can be performed within the first few days of life [3]. Thoracoscopic repair is a less invasive and cosmetically superior approach, and its surgical outcome does not significantly differ with that of the open approach [4, 5]. However, intrathoracic manipulation is challenging to perform in a narrow working space, as in that in neonates, and the esophageal tissue, particularly in the inferior esophageal segment, can easily tear due to fragility. Hence, the surgical indication of thoracoscopic repair is under debate. Moreover, pediatric surgeons must select the optimal surgical approach based on the characteristics of the patients. Weight is one of the criteria of thoracoscopic surgery, and some pediatric surgeons recommend thoracoscopic surgery only to patients weighing > 2000 g because the intrathoracic space of neonates weighing < 2000 g is extremely narrow for manipulation [6, 7]. In addition, at several institutions in Japan, patients weighing < 2000 g undergo open surgery [8, 9]. Nevertheless, there are only a few studies investigating the surgical outcomes of thoracoscopic surgery for EA/TEF in patients weighing < 2000 g. Moreover, some reports have recommended the use of thoracoscopic surgery in these patients [10].

Day of surgery is another risk factor. In other words, surgery for EA/TEF can be performed 24 h after stabilization [11]. Thus far, based on previous reports, primary repair at the age of ≥ 1 days has been the standard treatment [5, 8, 10]. However, emergency surgery for EA/TEF is occasionally performed because of respiratory failure or comorbidities of gastrointestinal atresia at the age of 0 day. In such a situation, not only TEF ligation but also primary repair is performed based on the institution’s policy [12, 13]. Nonetheless, to date, surgical outcomes based on the day of surgery after birth are unclear.

At our institution, thoracoscopic surgery for EA/TEF was introduced in August 2013. If the blind end of the upper esophagus is located cephalad to the clavicle, the operation is performed through a cervical incision. Esophageal banding and gastrostomy are performed in patients who cannot tolerate radical surgeries. For other patients with gross type C TEF, thoracoscopic primary repair without concomitant gastrostomy is performed [14]. These patients include the low-body-weight cases and emergency cases operated at 0 day because there was a lack of evidence to avoid thoracoscopic primary repair. We empirically considered that this procedure can be safely performed in these patients. Therefore, the current study aimed to investigate the effects of body weight and age at surgery on the outcomes of thoracoscopic primary repair for gross type C EA.

## Methods

This study retrospectively reviewed patients with gross type C EA/TEF who underwent primary repair from August 2013 to December 2022. Only patients who underwent thoracoscopic primary surgery were included in the analysis. Clinical data, including demographic characteristics of the patients, perioperative and postoperative information, and surgical complications based on the Clavien–Dindo classification, were collected from the medical records. Operation time included only the time of thoracoscopic surgery for EA/TEF but did not include the time of concurrent surgery. Patients weighing > 2 kg (group A) and those weighing < 2 kg (group B), and patients who underwent surgery at the age of 0 day (group C) and those who underwent surgery at the age of ≥ 1 day (group D) were compared. Quantitative variables were expressed as median and range. Qualitative variables were expressed as number and percentages. The Mann–Whitney U test was used to compare continuous variables, and the Fisher’s exact test was utilized to compare categorical variables. *P* values of < 0.05 were considered statistically significant. Statistical analyses were performed using Easy R (EZR) (Saitama Medical Center, Jichi Medical University, Saitama, Japan). This study was conducted based on the 1964 Declaration of Helsinki and its later amendments. The analysis used anonymous clinical data. This study was approved by the Institutional Review Board (Approval Number: 2023–0169). Patients were not required to provide a written informed consent. However, the opt-out method was applied to obtain consent for this study.

Surgical procedures for EA/TEF with the thoracoscopic approach

After intubation, the location of the TEF was confirmed using a fiber-optic bronchoscope. Patients were placed in the prone position, as described in a previous study [15]. A 5-mm camera port was inserted into the 5th intercostal space, and two 3-mm ports were inserted into the 4th and 6th intercostal spaces at the right chest. Manipulation was performed under a positive pressure pneumothorax of 4–8 mmHg. If lung expansion occurred, the pressure was temporarily increased to ≥ 5 mmHg after the anesthetist determined that the patient’s respiratory status was acceptable. Once the lung contracted, the pressure was immediately decreased to ≤ 4 mmHg. The TEF was ligated using 4–0 absorbable sutures. The proximal and distal segments were cautiously detached. The tips of the esophageal segment were opened and anastomosed using 6–8 needles with 5–0 polydioxanone RB-3 (PDS; Ethicon Inc., Somerville, NJ).

## Results

Forty-nine patients who had gross type C EA/TEF were identified during the study period. Four patients who underwent staged surgery (two patients with a long gap, one with unstable condition due to cardiac anomaly, and one weighing < 1000 g) and two patients who underwent surgery using the open cervical approach due to a distal fistula connected to the cranial side were also excluded. Forty-three patients who underwent thoracoscopic primary surgery without gastrostomy were included in this study. No cases involved conversion to open repair. Five patients had cardiac anomaly. However, none of the patients had cyanotic congenital heart disease. These patients were compared based on body weight at surgery. Nine patients were included in group A and thirty-four in group B. Table 1 shows the demographic characteristics of the patients. The median body weights at surgery were 1807 g in group A and 2617 g in group B. Table 2 shows the surgical outcomes between groups A and B. The two groups had similar surgical outcomes and the risk of postoperative complications such as chylothorax (greater than grade II), anastomotic leakage (greater than grade II), anastomotic stenosis requiring balloon dilatation, and gastroesophageal reflux requiring fundoplication.

**Table 1:** Characteristics of the patients compared based on body weight

	< 2000 g (*n* = 9)	≧2000 g (*n* = 34)	*P* value
Gender (male), *n* (%)	2 (22%)	21 (62%)	0.059
Gestational age ≧37 weeks, n (%)	**2 (22%)**	**30 (88%)**	0.00*
Weight at birth, g (range)	**1894 (1668–1958)**	**2651 (2020–3413)**	0.00*
Timing of surgery			
Day 0, *n* (%)	3 (33%)	4 (12%)	0.15
Day 1, *n* (%)	2 (22%)	18 (53%)	0.14
Day 2, *n* (%)	4 (44%)	9 (26%)	0.41
Day 3, *n* (%)	0 (0%)	3 (9%)	1.00
Weight at surgery, g (range)	**1807 (1668–1985)**	**2617 (2020–3325)**	0.00*
Cardiac anomaly, *n* (%)	1 (11%)	4 (12%)	1.00
Follow-up period, month (range)	36.7 (10.7–103.3)	52.2 (0.5–96.5)	0.57

Bold and asterisk “*” indicate statistically significant difference of *P* value under 0.05

**Table 2:** Surgical outcomes of the patients compared based on body weight

	< 2000 g (*n* = 9)	≧2000 g (*n* = 34)	*P* value
Operation time, min (range)	142 (106–267)	143 (73–295)	0.95
Blood loss, mg/kg (range)	0.5 (0–5.9)	0.4 (0–7.0)	0.82
Enteral feeding, day (range)	3 (1–31)	3 (3–7)	0.74
Anastomosis leakage, *n* (%)	0 (0%)	2 (6%)	1
Chylothorax, *n* (%)	2 (22%)	4 (12%)	0.59
Anastomotic stenosis requiring balloon dilatation, *n* (%)	2 (22%)	12 (35%)	0.69
GER requiring fundoplication, *n* (%)	0 (0%)	4 (12%)	0.56

Further, the patients were compared based on age at surgery. Seven patients were included in group C and thirty-six in group D. The reason for emergency surgery at the age of 0 day in group C was respiratory failure requiring ventilator management in four patients and comorbidities of duodenal stenosis/atresia in two patients. Table 3 shows the demographic characteristics of the patients. Table 4 shows the surgical outcomes between groups C and D. Intraoperative outcomes and postoperative complications except for anastomotic leakage were similar between the two groups. Two patients in group C, but none in group D, developed anastomotic leakage. Hence, the results significantly differed. The patients with anastomotic leakage improved with conservative therapy.

**Table 3:** Characteristics of the patients compared based on age at surgery

	Day0 (*n* = 7)	Day ≧1 (*n* = 36)	*P* value
Gender (male), *n* (%)	3 (43%)	20 (56%)	0.69
Gestational age ≧37 weeks	3 (43%)	29 (80%)	0.06
Weight at birth, g (range)	2454 (1668–2800)	2634 (1705–3413)	0.18
Age at surgery, day (range)	**0 (0–0)**	**1 (1–3)**	0.00*
Weight at surgery, g (range)	2454 (1668–2800)	252 1(1671–3325)	0.26
Cardiac anomaly, *n* (%)	1 (14%)	4 (11%)	1.00
Follow-up period, year (range)	63.1 (2.0–88.7)	45.0 (0.5–103.3)	0.86

Bold and asterisk “*” indicate statistically significant difference of *P* value under 0.05

**Table 4:** Surgical outcomes of the patients compared based on age at surgery

	Day0 (*n* = 7)	Day ≧1 (*n* = 36)	*P* value
Operation time, min (range)	135 (101–267)	147 (73–295)	0.79
Blood loss, mg/kg (range)	0.5 (0–1.6)	0.4 (0–7.0)	0.72
Enteral feeding, day (range)	3 (3–31)	3 (1–11)	0.21
Anastomosis leakage, *n* (%)	**2 (29%)**	**0 (0%)**	0.02*
Chylothorax, *n* (%)	1 (14%)	5 (15%)	1
Anastomotic stenosis requiring balloon dilatation, *n* (%)	1 (14%)	13 (36%)	0.40
GER requiring fundoplication, *n* (%)	2 (29%)	2 (6%)	0.12

Bold and asterisk “*” indicate statistically significant difference of *P* value under 0.05

## Discussion

Patients with EA/TEF are occasionally born with adverse conditions requiring surgery. These include low birth weight, unstable respiratory condition (which needs emergency ligation of the TEF), and cardiac anomaly. Pediatric surgeons must plan the surgical approach including the performance of thoracoscopic or open and primary or staged repair.

This study investigated the surgical outcome of thoracoscopic repair for gross type C TEF based on weight at surgery. The result was similar between patients weighing < 2000 g and those weighing > 2000 g. Thoracoscopic surgery for EA/TEF is still challenging to perform and has a significant learning curve associated with complications. Hence, surgeons must have sufficient skills and experience [8, 16, 17]. When performing thoracoscopic surgery for neonates with a low body weight, how small a space that can be safely operated in is an issue. Esophageal anastomosis can be tied with the extracorporeal knot-tying techniques. However, detaching around the esophageal pouch and suturing with the 5–0 polydioxanone RB-3 needle requires a specific amount of space. We decided that those who can perform esophago-esophageal anastomosis in less than 15 min on the simulator for neonatal esophageal atresia can be operators. In addition, surgeons with > 50 cases of experience in thoracoscopic repair are always present during surgery, either as an operator or as the first assistant. Based on our surgical outcomes, thoracoscopic primary repair was safely completed with no special surgical procedure. Therefore, manipulation in patients with low body weight is not risky for surgeons with sufficient skills and experience despite some challenges, and the incidence of complications did not increase. However, in our study, the lowest body weight observed was 1668 g, and it was unclear whether thoracoscopic primary repair was safe in smaller patients. Several reports have investigated the surgical outcomes of primary open repair in infants with extremely and very low birth weight (< 1500 g). However, there is no consensus regarding which approach is superior (primary vs. staged surgery). One study reported that body weight was not associated with an increased risk of surgical complications [18]. Another research recommended staged repair because primary repair increased the risk of surgical complications in patients weighing < 1500 g [19, 20]. The surgical indication of thoracoscopic surgery for patients weighing < 1500 g remains unclear based on previous reports. Therefore, surgeons should carefully select the surgical procedure, considering factors such as the patient’s ability to tolerate thoracoscopic surgery, comorbidities, the expertise of the surgeon and anesthesiologist, and the availability of appropriate instruments.

Further, our study compared patients according to the day of surgery. Patients with TEF occasionally require emergency ligation of the fistula. If a patient with the TEF is under positive ventilation due to respiratory distress, substantial amounts of gas are pumped into the stomach via the fistula. This leads to severe gastric and small bowel distention, thereby worsening respiratory condition due to chest compression and causing gastric perforation [12, 13]. Furthermore, comorbidities associated with gastrointestinal atresia can lead to abdominal distention and may result in respiratory failure. Decompression using a nasogastric tube is necessary to prevent respiratory failure in such cases, but it is impractical for gross type C TEF. Although surgery for TEF is typically performed semi-urgently, emergency surgery is required at birth for these cases due to the high risk of sudden and fatal deterioration of respiratory conditions. Even when surgery is performed on the day of birth, the patient’s respiratory and general condition remains stable. If the general and respiratory condition is unstable, thoracoscopic repair is not selected. Our study included patients who underwent thoracoscopic primary repair at the age of 0 day due to respiratory support requirements or comorbidities such as duodenum atresia/stenosis. Results showed that emergency surgery at the age of 0 day increased the risk of anastomotic leakage compared with surgery performed at the age of > 1 day. In addition, the surgical outcomes were compared between patients who underwent surgery at the age of 1 day and those who underwent surgery at the age of ≥ 2 days. However, the results did not significantly differ. The whole body of newborns is swollen just after birth, and tissue edema leads to anastomosis fragility. Hence, there is a high risk of suture failure whether the procedure is performed via thoracoscopy or thoracotomy. To prevent this issue, only the TEF can be initially ligated, and radical surgery can be performed after a while. Anastomotic leakage generally delays oral intake until confirming the absence of leakage on upper gastrointestinal series. However, in our cases, the patients’ condition improved with conservative therapy, and enteral feeding had been continually facilitated via the nasogastric tube inserted during radical surgery. The prognosis remained the same in our study, but the use of the thoracoscopic primary approach at the age of 0 day must be performed with extra caution.

In our case series, the incidence of postoperative chylothorax was higher compared to the previously reported 3% [21]. This can be attributed to lymph vessel injury during cauterization of the azygos vein, as described in our previous report [22]. Following improvements in manipulation techniques, the incidence of postoperative chylothorax decreased.

This study had several limitations. That is, it was a retrospective, nonrandomized, single-institution study, and the sample size was small. Thoracoscopic primary surgery for patients weighing < 2000 g and those who underwent surgery at the age of 0 day was performed by one skilled pediatric surgeon. Accordingly, further multicenter prospective studies with larger sample sizes and multiple surgeons should be performed to accurately determine the surgical outcomes.

## Conclusion

Thoracoscopic primary repair is safe for patients with low body weight (1500–2000 g). By contrast, emergency surgery at the age of 0 day is associated with a risk of anastomotic leakage. Hence, thoracoscopic surgery should be performed with more caution at the age of 0 day.

## Data Availability

No datasets were generated or analysed during the current study.

## References

[1] Lupo PJ, Isenburg JL, Salemi JL, Mai CT, Liberman RF, Canfield MA et al (2017) Population-based birth defects data in the United States, 2010–2014: a focus on gastrointestinal defects. Birth Defects Res 109:1504–1514 29152924 10.1002/bdr2.1145 PMC5915361

[2] Pedersen RN, Calzolari E, Husby S, Garne E (2012) Oesophageal atresia: prevalence, prenatal diagnosis and associated anomalies in 23 European regions. Arch Dis Child 97:227–232 22247246 10.1136/archdischild-2011-300597

[3] van der Zee DC, Bagolan P, Faure C, Gottrand F, Jennings R, Laberge JM et al (2017) Position paper of INoEA working group on long-gap esophageal atresia: for better care. Front Pediatr 5:63 28409148 10.3389/fped.2017.00063 PMC5374143

[4] Way C, Wayne C, Grandpierre V, Harrison BJ, Travis N, Nasr A (2019) Thoracoscopy vs thoracotomy for the repair of *esophageal* atresia and tracheoesophageal fistula: a systematic review and meta-analysis. Pediatr Surg Int 35:1167–1184 31359222 10.1007/s00383-019-04527-9

[5] Yang S, Wang P, Yang Z, Li S, Liao J, Hua K et al (2021) Clinical comparison between thoracoscopic and thoracotomy repair of gross type *C**esophageal* atresia. BMC Surg 21:403 34809633 10.1186/s12893-021-01360-7 PMC8607600

[6] Dingemann C, Ure BM (2013) Minimally invasive repair of esophageal atresia: an update. Eur J Pediatr Surg 23:198–203 23720207 10.1055/s-0033-1347914

[7] Holcomb GW, Rothenberg SS, Bax KM, Martinez-Ferro M, Albanese CT, Ostlie DJ et al (2005) Thoracoscopic repair of esophageal atresia and tracheoesophageal fistula: a multi-institutional analysis. Ann Surg 242:422–428 16135928 10.1097/01.sla.0000179649.15576.db PMC1357750

[8] Okuyama H, Tazuke Y, Ueno T, Yamanaka H, Takama Y, Saka R et al (2018) Learning curve for the thoracoscopic repair of esophageal atresia with tracheoesophageal fistula. Asian J Endosc Surg 11:30–34 28718991 10.1111/ases.12411

[9] Yamoto M, Urusihara N, Fukumoto K, Miyano G, Nouso H, Morita K et al (2014) Thoracoscopic versus open repair of esophageal atresia with tracheoesophageal fistula at a single institution. Pediatr Surg Int 30:883–887 25052256 10.1007/s00383-014-3554-2

[10] Son J, Jang Y, Kim W, Lee S, Jeong JS, Lee SK et al (2021) Thoracoscopic repair of esophageal atresia with distal tracheoesophageal fistula: is it a safe procedure in infants weighing less than 2000 g? Surg Endosc 35:1597–1601 32323019 10.1007/s00464-020-07538-z PMC7222104

[11] Wang B, Tashiro J, Allan BJ, Sola JE, Parikh PP, Hogan AR et al (2014) A nationwide analysis of clinical outcomes among newborns with esophageal atresia and tracheoesophageal fistulas in the United States. J Surg Res 190:604–612 24881472 10.1016/j.jss.2014.04.033

[12] Malone PS, Kiely EM, Brain AJ, Spitz L, Brereton RJ (1990) Tracheo-oesophageal fistula and pre-operative mechanical ventilation. Aust N Z J Surg 60:525–527 2357177 10.1111/j.1445-2197.1990.tb07419.x

[13] Maoate K, Myers NA, Beasley SW (1999) Gastric perforation in infants with oesophageal atresia and distal tracheo-oesophageal fistula. Pediatr Surg Int 15:24–27 9914349 10.1007/s003830050504

[14] Nakagawa Y, Uchida H, Shirota C, Tainaka T, Sumida W, Makita S et al (2023) Preoperative contrast examinations help determine the appropriate cervical approach for congenital gross Type C esophageal atresia: a report of two cases. Am J Case Rep 24:e938723 37309107 10.12659/AJCR.938723 PMC10274229

[15] Shirota C, Tanaka Y, Tainaka T, Sumida W, Yokota K, Makita S et al (2019) Therapeutic strategy for thoracoscopic repair of esophageal atresia and its outcome. Pediatr Surg Int 35:1071–1076 31399810 10.1007/s00383-019-04541-x

[16] Lee S, Lee SK, Seo JM (2014) Thoracoscopic repair of esophageal atresia with tracheoesophageal fistula: overcoming the learning curve. J Pediatr Surg 49:1570–1572 25475795 10.1016/j.jpedsurg.2014.04.016

[17] Guo Y, Hinoki A, Deie K, Tainaka T, Sumida W, Makita S et al (2023) Anastomotic time was associated with postoperative complications: a cumulative sum analysis of thoracoscopic repair of tracheoesophageal fistula in a single surgeon’s experience. Surg Today 53:1363–1371 37087700 10.1007/s00595-023-02687-9

[18] Schmidt A, Obermayr F, Lieber J, Gille C, Fideler F, Fuchs J (2017) Outcome of primary repair in extremely and very low-birth-weight infants with esophageal atresia/distal tracheoesophageal fistula. J Pediatr Surg 52:1567–1570 28554817 10.1016/j.jpedsurg.2017.05.011

[19] Petrosyan M, Estrada J, Hunter C, Woo R, Stein J, Ford HR et al (2009) Esophageal atresia/tracheoesophageal fistula in very low-birth-weight neonates: improved outcomes with staged repair. J Pediatr Surg 44:2278–2281 20006009 10.1016/j.jpedsurg.2009.07.047

[20] Ritz LA, Widenmann-Grolig A, Jechalke S, Bergmann S, von Schweinitz D, Lurz E et al (2020) Outcome of patients with esophageal atresia and very low birth weight (≤ 1500 g). Front Pediatr 8:587285 33282800 10.3389/fped.2020.587285 PMC7705242

[21] Lal DR, Gadepalli SK, Downard CD, Ostlie DJ, Minneci PC, Swedler RM et al (2017) Perioperative management and outcomes of esophageal atresia and tracheoesophageal fistula. J Pediatr Surg 52:1245–1251 27993359 10.1016/j.jpedsurg.2016.11.046

[22] Shirotsuki R, Uchida H, Tanaka Y, Shirota C, Yokota K, Murase N et al (2018) Novel thoracoscopic navigation surgery for neonatal chylothorax using indocyanine-green fluorescent lymphography. J Pediatr Surg 53:1246–1249 29486888 10.1016/j.jpedsurg.2018.01.019

